# Exploring the Coordination of Cancer Care for Teenagers and Young Adults in England and Wales: BRIGHTLIGHT_2021 Rapid Qualitative Study

**DOI:** 10.3390/cancers17233874

**Published:** 2025-12-03

**Authors:** Elysse Bautista-Gonzalez, Rachel M. Taylor, Lorna A. Fern, Julie A. Barber, Jamie Cargill, Rozalia Dobrogowska, Richard G. Feltbower, Laura Haddad, Nicolas Hall, Maria Lawal, Martin G. McCabe, Sophie Moniz, Louise Soanes, Dan P. Stark, Cecilia Vindrola-Padros

**Affiliations:** 1 Centre for Nurse, Midwife and AHP Led Research (CNMAR), University College London Hospitals NHS Foundation Trust, London NW1 2PG, UK; 2 Cancer Clinical Trials Unit, University College London Hospitals NHS Foundation Trust, London NW1 2PG, UK; 3 Department of Statistical Science, University College London, London WC1E 6BT, UK; 4 Managed Service Network for Children and Young People with Cancer in Scotland, Kings Cross Hospital, Dundee DD3 8EA, UK; 5 Rapid Research Evaluation and Appraisal Lab (RREAL), Department of Targeted Intervention, University College London, London WC1E 6BT, UK; 6 Leeds Institute for Data Analytics, School of Medicine, University of Leeds, Leeds LS2 9JT, UK; 7 Patient Representative, BRIGHTLIGHT Young Advisory Panel (YAP), UK; 8 Division of Cancer Sciences, The University of Manchester, Manchester M13 9PL, UK; 9 Teenage Cancer Trust, London WC1V 7AA, UK; 10 Leeds Institute of Medical Research at St James’s, Leeds LS9 7TF, UK

**Keywords:** cancer, young people, teenagers, young adults, BRIGHTLIGHT, coordination, collaboration, leadership, model of care

## Abstract

Background: Commissioning of ‘joint care’ across teenage and young adult (TYA) principal treatment centres (PTC) and regional designated hospitals was introduced to enable cancer care closer to home, while providing support through the TYA multidisciplinary team. We aimed to explore the processes being used to enable inter-organisational collaboration under joint care models through rapid ethnography. Methods: Healthcare professionals in TYA PTCs in England and Wales between June 2022 and December 2023 were identified by the TYA lead in each PTC as delivering TYA cancer care. Semi-structured interviews were conducted virtually or by telephone based on the structuration model of collaboration proposed by D’Amour. Data were analysed against the model through framework analysis. Results: Our study highlighted variation across the different dimensions of inter-organisational collaboration. We found that healthcare professionals delivering TYA cancer care were working toward a shared goal but this was not always achieved. Social interaction between professionals was required to develop relationships and trust, but opportunities for social interaction were not regularly available. Processes for sharing information were not streamlined, so there were instances when information could not be shared between organisations. Interventions to achieve coordinated care, such as an outreach team, supported the delivery of joint care but these were not available in every region. While there were some levels of leadership within aspects of services, there were limited examples nationally or across geographical regions, which hindered the development of coordinated care. Conclusions: Coordination of care is mostly developing; however, the shared vision and goals dimension did achieve full active collaboration. The implementation of a service specification will address regional leadership requirements, but resources are required to extend the delivery of interventions to support coordination and collaboration, allowing the commissioned model of care to be delivered safely.

## Simple Summary

A joint care model for teenage and young adult (TYA) cancer patients was introduced across principal treatment centres (PTCs) and designated hospitals to provide care closer to home with support from TYA teams. This study explored how healthcare teams in England and Wales collaborated under this model using interviews with professionals involved in TYA cancer care. Analysis was based on D’Amour’s model of collaboration. The findings showed variation in how well organisations worked together. While staff generally shared the same goals, collaboration was not always fully achieved. Opportunities for relationship-building and trust were limited, and information sharing between organisations was inconsistent. Outreach teams helped deliver joint care, but these were not available in all regions. Leadership to support coordination was also lacking in some areas. Although some aspects of collaboration were developing well, others needed improvement. National commissioning supported by additional resources is needed to fully implement coordinated and effective joint care.

## 1. Introduction

Cancer care in the United Kingdom (UK) has evolved from the standard children and adult model of healthcare delivery to include specific services for teenagers and young adults (TYA), for those aged 13–24 years at diagnosis. In England, specific services for TYA were introduced in thirteen geographical regions based on guidance published in 2005 by the National Institute for Health and Care Excellence (NICE) [1]. This consisted of the TYA Principal Treatment Centre (PTC), comprising a bespoke TYA unit in a central hospital and workforce skilled to deliver young people’s cancer care. The TYA PTCs incorporated the core components of the NICE guidance services and were designed to reflect the needs of the local population. This included the age eligibility for admission, which could vary between 13 and 24 years [2]. The NICE recommendation was for all those aged 19 or under to be referred to an ‘age-appropriate’ unit and those aged 19–24 years to have the choice on whether to stay in an adult cancer unit closer to home or travel to the TYA PTC. To ensure young people aged 19–24 were not negatively impacted if they chose to receive care in a local adult cancer unit, a process of designation, known as Designated Hospitals (DH), was introduced so young people would have some aspects of age-appropriate care, if not the full comprehensive package that was proposed as being delivered in the PTC. Similar models of care have since been implemented in Scotland and Wales [3].

The evidence underpinning these recommendations was limited and based mostly on expert consultation and patient advocacy [4]. The first comprehensive evaluation of UK TYA services was undertaken between 2012 and 2019 by the BRIGHTLIGHT Study [5]. This included identifying the competencies of the TYA workforce delivering care through a Delphi survey [6]; determining patient and caregiver outcomes and experiences related to place of care in a longitudinal cohort study [7,8,9,10,11]; and understanding the culture in the environments where care was delivered through a case study [12,13,14]. The results of the cohort study indicated patient-reported outcomes were lower for young people who received some care in the TYA PTC and the rest elsewhere, in either a children’s or adult’s cancer service [7,8]. However, by 2010, less than half the TYA PTCs were well established, with the remaining PTCs developing their infrastructure and establishing collaborations across their geographical regions. The poorer outcomes in those receiving some TYA PTC care were therefore surmised as being due to uncoordinated care seen in an embryonic culture. Developments in organisational culture over time could have led to the establishment of better processes for collaboration and coordination of care, producing similar patient-reported outcomes irrespective of where care is delivered. In this study BRIGHTLIGHT_2021, we aimed to explore the processes being used to enable inter-organisational collaboration under joint care models for TYA.

Models of joint care, as outlined in the TYA service specification [15], and other forms of inter-organisational collaboration have been identified as essential for facilitating care delivery across various provider networks [16,17]. Inter-organisational collaboration is acknowledged as a vital component in ensuring seamless care delivery. It involves complex interactions and processes among multiple organisations sharing a common goal, including the coordination of patient transfers between different providers, professionals, and healthcare settings [17,18]. Despite the importance of these collaborations, there is limited understanding of how daily inter-organisational practices are executed and sustained within TYA networks [16,19].

## 2. Methods

### 2.1. Study Design

BRIGHTLIGHT_2021 was a mixed methods study comprising a cross-sectional survey of young people newly diagnosed with cancer in the UK and qualitative study involving healthcare professionals. This paper reports the qualitative aspect of BRIGHTLIGHT_2021, based on rapid ethnography using semi-structured interviews [20,21]. In this study, we explored the coordination of TYA cancer care between the TYA PTC and DH in England and Wales. The analysis was guided by a conceptual framework informed by previous work on collaborative relationships in healthcare contexts developed by D’Amour et al. [17], as well as adaptations of this network we have made in previous research [18].

### 2.2. Patient and Public Involvement

The BRIGHTLIGHT program (both 2012 and 2021) was co-produced with young people who have lived experience of a cancer diagnosis and treatment: the Young Advisory Panel (YAP). Their interpretation of the 2012 BRIGHTLIGHT program of work provided the justification for the 2021 protocol [22]. While this qualitative aspect of the study focused on the healthcare professional perception, members of the YAP reviewed study documentation and the interview schedule.

### 2.3. Participants and Setting

We sought to open the study in all TYA PTCs in England and include the TYA services in Scotland and Wales. As each site was opened to recruitment, one of the clinical leads for the TYA service met with the researcher to describe how their service operated and to identify healthcare professionals across their care network, including several DHs, who could be approached to participate. We sought to recruit a purposive sample of staff representing the professions in the multi-disciplinary team (MDT) from the TYA PTC and DH. Staff were invited to participate through email and if they consented to take part, the interview was organised at a time convenient for them. Up to ten professionals at each site were invited to participate.

### 2.4. Data Collection

Data were collected from June 2022 to December 2023 through semi-structured interviews either in person, on Microsoft Teams or over the phone. Interviews were conducted by experienced researchers who had no previous relationship with the interviewees and were informed by a topic guide based on the framework proposed by D’Amour et al. (Supplemental File). The interviews were recorded and transcribed verbatim. To facilitate constant comparison, i.e., include questions in subsequent interviews based on emerging findings, RREAL sheets were used [21].

### 2.5. Analysis 

Data collection and analysis were carried out in parallel. The categories used in the RREAL sheets were based on the questions included in the interview topic guide, maintaining flexibility to add categories as the study was progressing [23]. Data from the interviews were imported into NVivo and analysed using framework analysis [24,25,26], using D’Amour’s structuration model. defined as the process of giving structure or organisation to care and focuses on relationships between professionals and the interactions they have within the healthcare system. The model considers four dimensions of collaboration operationalised into ten indicators (Table 1) [17]. Indexing and charting were conducted by four researchers (CVP, SM, NH, RD) and mapping and interpretation was undertaken independently by an additional four researchers (CVP, EBG, RMT, LAF). Finally, results from the qualitative analysis were quantified and scored, as originally proposed by D’Amour et al. [17]. each of the 10 indicators (Table 1) were assigned a score of 1 to 3 depending on the level of achievement of the indicator of collaboration (3 = active collaboration, 2 = developing collaboration, 1 = latent or potential collaboration). This was undertaken independently by three members of the research team (EBG, RMT, LAF) and the mean score on each indicator was used to portray results using a Kiviat graph [27]. The graph helps visualise the gaps between optimal collaboration processes and the current situation.

## 3. Results

The study was conducted in ten TYA PTCs in England and Wales (n = 11). It was not possible to open the study in three TYA PTCs in England or anywhere in Scotland due to the ongoing impact of the pandemic and workforce capacity issues. Forty-one interviews were conducted with participants in the PTCs, six in the DHs and 11 with staff working across PTCs and DHs. Participants represented doctors (n = 18), nurses (n = 23), other healthcare professions (n = 7), managers (n = 6) and youth support coordinators (n = 4). The total interview time was 1567 min, with a median of 30 min and a range of 11–48 min.

The framework analysis highlighted that while PTCs and DHs had shared goals for patient-centred care and engaged in some collaborative practices, significant challenges in formalisation, information exchange, governance, and leadership hindered the consistency and effectiveness of their collaboration. The findings are summarised in Table 2 and presented in detail according to the four dimensions in the subsequent text, illustrated by relevant quotes.

### 3.1. Shared Goals and Vision

Healthcare teams delivering TYA cancer care acknowledged the unique needs of young people and emphasised the importance of holistic support, aiming to help patients maintain normalcy beyond cancer diagnosis into survivorship. The members of the MDT were proactive in communication and collaboration with DH, ensuring robust support networks and continuity of care, demonstrating a cohesive effort to meet shared objectives effectively. This included delivering psychosocial support and coordinating with local teams to minimise patient travel, highlighting a joint approach to palliative care and symptom management.

“I’m all about collaborative working. We’ve all got different skills, haven’t we? And we can’t all know everything. So, it’s about getting everybody together and working jointly without any ego. Because a lot of people have egos. Getting rid of that and just putting the young person in the middle.”

(Clinical Nurse Specialist)

Choice of care was influenced by patients, however, sometimes staff views impacted this. Some staff prioritised local treatment while others thought it was best for patients to be treated in the PTC. In some regions, agreements for joint care were non-existent or it was limited due to aspects of care, e.g., clinical trials needing the patient to receive services only in the PTC. Professionals in DHs sometimes mentioned that there was no established joint care model.

“And doing things like, you know, if somebody is being treated here but I’m in touch with the teams nearer to them if they need line care or medications and things, to avoid them having to travel. Just trying to make things as easy for them [young people] as possible, wherever they are.”

(Clinical Nurse Specialist)

PTC and DH delivering TYA cancer care demonstrated shared goals centred on providing quality care for young people with cancer. This was evidenced by their commitment to care through an age-appropriate environment and ensuring equal quality of care regardless of treatment location. While patients faced different pathways to care, TYA cancer care strove to offer consistent care experiences both at the PTC and DH. When not in the PTC, health professionals in the DH aimed to ensure the same nurse delivered care to young people or implemented mechanisms for consistent care, for example, accompanied the young person into the adult ward, assigned a particular day for young people or put aside rooms for them. Both PTCs and DHs aimed to guide patients effectively through their treatment journey either through the outreach coordinator, disease specific clinical nurse specialist (CNS) or TYA CNS.

“So, the most important aspects of care are the- I think it’s the designated spaces for young people, which make them feel safe and at home, like it’s not a hospital and they’re not surrounded by old people. Because it’s frightening for them. And alongside that, obviously, it’s the staff that, who are used to looking after young people. Because it’s a different entity.”

(Clinical Nurse Specialist)

“The idea is that most of the services we provide within the- our principal treatment center can be provided on an outreach basis.”

(Consultant Haematologist)

TYA cancer care teams aimed to deliver patient-centred care through needs assessments and follow-up delivered in an informal format. This environment allowed young people to build and maintain a relationship with their healthcare provider and thus feel safe. TYA healthcare professionals were aware of the difficulties patients went through when having to choose place of care at the age of 18 and thus catered to their specific needs by providing a detailed explanation of the place of care and their differences. Despite efforts to deliver patient-centred care, according to healthcare professionals, there were certain patients who experienced challenges in receiving patient-centred cancer care. For example, in DHs, patients faced isolation due to lack of peer interactions, which usually took place at the PTC or by patients being secured in their rooms to avoid cross-infection. Additionally, in both the PTCs and DHs, there were staff shortages and high turnover. DHs were particularly impacted by this due to having fewer TYA trained staff and relied on the outreach support team to smooth transitions between the PTC and DH and establish local peer support.

“And just our general kind of informality with the teenagers is good. I think we still give them an air of professionalism and that’s important because you need to have some reassurance that, you know, that what they’re receiving is correct, accurate and so on. But it’s doing it all with a level of informality to try and, you know, take the scary nature out of, or the serious nature out of what they’re having to go through […] I think we adapt to patient-centered care very accurately. “

(Clinical Nurse Specialist)

From the mental health perspective, there was a lack of support for young people in DH due to lack of resources. Where this could be augmented with peer support, this was also not available as it only occurred at the PTC. Furthermore, mental health services were only provided after the cancer diagnosis and did not address pre-existing long-term mental health conditions.

“Has to be kind of based around their- any emotional distress related to treatment, or to diagnosis or treatment. We aren’t able to support with pre-existing or kind of long term severe and enduring mental health issues.”

(Clinical Psychologist)

“No outreach facilities for things like psychological support or specific physio, occupational therapy support for TYAs.”

(TYA Clinical Lead)

### 3.2. Internalisation

Trust was built through structured social interaction. Relationships were highly valued as an indicator for building trust and establishing effective collaborations between the PTC and DH. Both organisations recognised the importance of these relationships for effective patient transfer and joint care. Some relationships were built quicker than others, due to the incidence and prevalence of disease and repetition of encounters. However, healthcare professionals reported few opportunities to socially encounter their colleagues.

Those in the DH described an acute problem with lone workers and part-time staff, due to its impact on relationship building, objective completion, and lack of supervision. Similarly, some PTCs had increased their capacity and infrastructure within an organisation, and this had also caused a negative impact in forming relationships with colleagues that were no longer in close proximity. Although PTCs faced part-time staff coordination issues, they had a wider TYA team for support, so there was less impact on communication and coordination of care.

Due to the lack of mutual acquaintance, healthcare professionals in the DH found it challenging to get information about patient updates or clinical decisions by email and lacked representation in crucial PTC meetings. Similarly, professionals from the PTC held on to patients as long as possible to avoid the possibility of losing the patient and going back and forth not knowing if the patient would receive care in the DH. For example, transferring patients back to the DH when they turned 18 was infrequent and thus due to lack of acquaintance and a higher perceived sense of control over the patient’s outcome, it was preferred to keep the patient in the PTC.

Nonetheless, there were several factors that hindered the transitioning process: patients and family members had the perception of being transferred to second class care; there was no official transition protocol; clinicians decided patients needed to be in the PTC due to clinical reasons; geographical variation in access; and preference of scans and tests being done at the PTC rather than locally. In consequence, lack of mutual acquaintanceship and trust also impacted on a young person’s choice of place for care.

“While teams in the DH are good at remembering to communicate with PTC, the PTC is not necessarily good at communicating with the DH.”

(Clinical Nurse Specialist)

“I think my team works really well with knowing what each other’s roles are and how they cross over. So we’ve got the youth support coordinators, who are youth background- Myself, obviously, I was a nurse and have been for too many years. The social workers from [name of charity] […] They do have such varied backgrounds as well, so I think it’s all about communication, isn’t it? And we all have different things to offer the young person, but holistically we can do that really well, I think.”

(Clinical Nurse Specialist)

### 3.3. Formalisation

Young people were identified and discussed at TYA MDT meetings. Examples were reported where the TYA MDT contacted the outreach team when a young person was identified with suspected cancer. Once confirmed, the outreach worker was involved, offering a holistic needs assessment and presenting different care pathways. However, both the PTC and DH struggled with communication and coordination issues and at times there were blurred lines as to who had precise responsibilities.

Information sharing between PTCs and DHs was facilitated by TYA colleagues at the PTC. They shared information through formal information technology (IT) portals, email, MDT minutes, phone or face-to-face. However, some DH healthcare teams found it challenging to receive clinical communications, patient status updates or clinical decisions by email and were not always represented in crucial PTC meetings. Moreover, there was no standardisation between the DH and the PTC in the processes for recording and tracking newly diagnosed young people, so this was localised and ad hoc, impacting on the coordination of care.

After treatment, patients were followed heterogeneously upon discharge. Sometimes they were cared for in the community and sometimes in the hospital. Time for follow-up also varied from 1 to 2 years after treatment ended and was conducted in person, over the phone or via text in an unstandardised manner.

The presence of partial or inconsistent information systems indicated that there was scope for improvement in creating a common, efficient infrastructure for collecting and sharing information. For example, staff employed by charities were excluded from communication channels through not being able to access electronic health records or not receiving emails as they did not have an NHS email account (data protection regulations require patient identifiable information to pass from NHS-to-NHS email addresses only). Consequently, there were safety issues as the DH teams were not always informed about all aspects of the young person’s care. This could negatively impact their care if, for example, they required admission to the emergency department at the DH. Similarly, in some DH there were separate systems for dispensing paediatric and adult chemotherapy and not everyone had access to both, resulting in a long time for the pharmacist to calculate individualised dosage.

“I think we communicate with all of our colleagues within the trust in a productive and supportive way. I think, obviously, there are opportunities and sometimes where communication breaks down and that’s to be identified and to try and work upon. But I feel everyone is very accepting and understanding of the complexities of the teenage and young adult patients, so that’s, has always worked well.”

(Clinical Nurse Specialist)

### 3.4. Governance

There was no unified and clear leadership structure that effectively drove collaboration between PTC and DH for patients in joint care, adult or children wards. Leadership was inherent to the place of care. For example, outreach nurses identified and followed up patients, but they were not in charge of coordinating the full continuum of care. Similarly, a disease-specific CNS led the young person’s appointments and their blood tests, while the TYA CNS supported their journey. Alternatively, named consultants coordinated care in the young person’s chosen place of care. Hence, coordination of care was split between stakeholders within their place of care. If patients chose joint care, there was no established model of coordination nor leadership, not even for joint clinical decision-making as hospitals had their own site-specific MDTs.

“As CNSs are funded by TCT [we] do get together to share info about what is happening with which patient and who problems maybe arising. Tuesday psychosocial MDT, go through their tumor specific MDT for diagnosis and treatment discussion. Then they come to TYA, holistic—medics, lead nurse, dietician, physio, young lives versus cancer.”

(Youth Support Coordinator)

“There is also a weekly psychosocial MDT which is led by the nursing key workers […] also a fortnightly end-of-treatment MDT to discuss survivorship and long-term follow-up issues.”

(Consultant Medical Oncologist)

There were few joint clinical activities between health professionals working either at the PTC or DH. TYA MDT meetings became the central channel for communication and making professional acquaintances, but these were not standardised and sometimes PTC and DH timetables clashed, limiting the avenue for discussion. Furthermore, virtual communication as a consequence of COVID-19 pandemic restrictions, decreased face-to-face interactions, resulting in outreach nurses being based at the PTC or their work had shifted to working from home. This negatively impacted on the coordination of care.

“Online Teams meetings have helped to facilitate MDT representation from people in different locations as “you’d never get all of those people into a room”

(Consultant Clinical Psychologist)

“It’s [TYA MDT] often very much led, presented and explained to the TYA service and the head of TYA. Obviously when we reach the end of the clinical list and we come to the psychosocial list, most of the consultants from different places will go. And our head of TYA will then take over from a psychosocial perspective just to kind of act as a, almost like a chairperson I guess and just keep it flowing. But it’s a relatively open floor for, you know, formal and informal kind of chat, discussion and so on.”

(Clinical Nurse Specialist)

**Figure 1:**
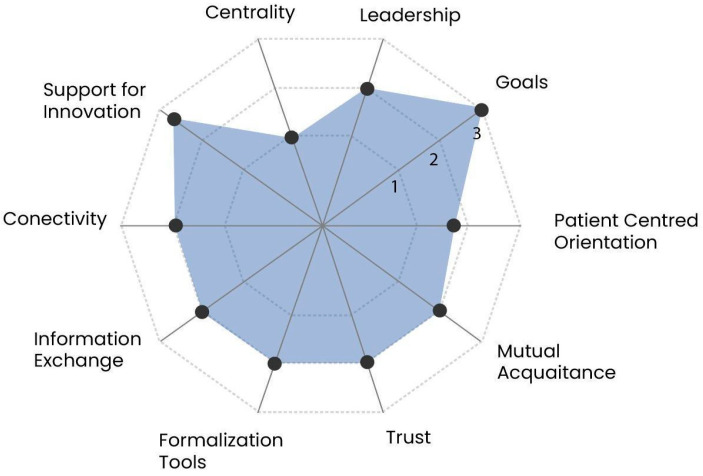
Kiviat graph mapping the collaboration between PTC and DH. The score of 1 to 3 was assigned to each indicator on how well it had been achieved, with centrality scoring the lowest and goals scoring the highest. The graph visualises where coordination is optimised and where it requires further strengthening.

The study sought to explore the current state of inter-organisational collaboration in the context of TYA cancer care. The analysis of the collaboration and coordination of care between the PTC and DH indicated that active collaboration was achieved for the ‘Goals’ indicator in the ‘Shared Goals and Vision’ dimension, whereas the ‘Centrality’ indicator in the ‘Governance’ dimension required further strengthening (Figure 1). The remaining eight indicators were rated as developing collaborations [22]. All healthcare professionals delivering TYA cancer care were working toward a shared goal, but this was not always achieved. Social interaction between professionals was required to develop relationships and trust (essential for establishing joint care) but there were few opportunities for social interaction. Processes for sharing information were not always in place so information was not always shared between organisations. Finally, while there were some levels of leadership within aspects of services, there were limited examples at a national scale or across geographical regions, which hindered the development of coordinated care.

## 4. Discussion

Coordinated care is defined as “the deliberate organization of patient care activities between two or more participants (including the patient) involved in a patient’s care to facilitate the appropriate delivery of healthcare services. Organizing care involves the marshalling of personnel and other resources needed to carry out all required patient care activities and is often managed by the exchange of information among participants responsible for different aspects of care.” [28]. Coordinated care is important because it improves the processes of care resulting in improved outcomes, for example, fewer hospitalisations and visits to the emergency department [29]. The Agency for Healthcare Research and Quality noted in the Care Coordination Measure Atlas that it was the responsibility of the system of care (i.e., the NHS) to integrate personnel, information and other resources to enable coordinated care. Furthermore, a review of the effectiveness of interventions to improve coordination noted these were mostly developed to improve communication and these were in the main led by nurse or patient navigators. There were no interventions identified that were led by physicians [29].

This is reflected in the way cancer care for children and subsequently TYA cancer care has developed in the UK. One of the main contributions of this study is its focus on care coordination through a network of providers and the analysis of changes in care delivery over time. The concept of joint care for TYA stems from the model of shared care implemented in the UK for children’s services in the 1970/80s, where care was shared across the children’s principal treatment centre and a paediatric oncology shared care unit (POSCU) [30]. Centralising care improved the delivery of treatment and reduced morbidity and mortality [31]. Through reducing the travel burden to families, shared care enabled delivery of care closer to home [32]. However, the staff in the POSCU did not have the knowledge or the skill to take overall care for children with cancer and, therefore, mid-1980s, a specialist nursing workforce was introduced who were the interface between care in the specialist children’s oncology units and other environments of care. The paediatric outreach oncology nurse specialists (POONS), who were initially employed to support terminally ill children, were a link between centralised services, care closer to home and primary care [32]. The success of POONS in improving outcomes supported a national implementation of the role beyond terminal illness, which helped streamline services [30].

Centralising health services for rare diseases or a minority population ensures that there is a critical mass of patients so the healthcare team gain expertise [33]. Centralised care was partially advocated for TYA in 2005 in national guidance [1], and the subsequent model of care spanning across a network rather than a specific hospital was commissioned in 2023 [15,34,35]. This aligns with the UK government’s aspiration for care closer to home wherever possible [36,37]. For young people this is important so they are able to maintain as much normalcy in their lives as possible during their journey through this difficult developmental stage. This supports joint care as specified in the service specification but this needs to be with the caveat that young people are in a supportive environment where they are safe and there are mechanisms in place for care to be coordinated.

The idea of having someone to ‘facilitate timely access to quality cancer care that meets cultural needs and standards of care’ [38], in the UK was packaged as the keyworker (usually a clinical nurse specialist). In other countries, such as North America, the patient navigator role has been established [39]. Patient navigators play an important role in the search for equitable care for all patients with cancer [40]. In comparison with coordination of care, case management, social work or advocacy, patient navigation programs aim to reduce health inequalities by considering the patient’s context and perspective [41,42,43,44,45]. Navigators have been shown to be particularly effective in countries where specialist TYA services cover a large geographical region. The patient navigator reduced logistical and administrative hurdles associated with accessing care, taking the traditional role of coordinating communication between the medical team, building networks and relationships to advocate for better patient outcomes. Most importantly, the navigator provided a TYA aspect to “coordinate services to ensure patients do not fall through the cracks”. It is the young person’s knowledge rather than cancer that enables this [46,47,48].

In the current study, more coordinated care was often attributed to the outreach team. Mostly these were nurses but, in some instances, they were youth workers. The outreach team had the remit of empowering teams in the DH to be confident in communicating and treating young people, a skill that healthcare professionals working in adult oncology have reported as being challenging [49]. The outreach nursing role was introduced after the 2012 BRIGHTLIGHT case study was conducted, so the benefit was not explored in our earlier evaluation. However, a separate pilot evaluation of the outreach model of care indicated more young people were identified in the local hospitals and they could be linked to the age-appropriate services in the TYA PTC. The evaluation also confirmed outreach staff provided better coordination and communication between services that were more holistic [50].

Previously, the 2012 BRIGHTLIGHT case study showed that culture was dependent on leadership in bringing the whole team together. It was especially important for TYA leaders in the DH because the absent or limited TYA services meant there was a need to engage a wider workforce than the PTC who were more established. These leads were characterised by having enthusiasm and passion, and the drive to develop relationships with their adult colleagues working in tumour-specific specialties, often in silos. Challenges emerged when the service relied upon a single TYA lead in the DH. When the ‘leader’ left, there was uncertainty and disjointed care [12,14]. In this current study, although there was a continuous assessment of barriers in cancer care and tailored approaches to mitigate them, there was no mention of central authorities to coordinate care or protocols. D’amour et al. noted that the governance dimension was crucial because without leadership, such patient-centred efforts became fragmented and hindered collaborative practices [17]. In the current study, regional leadership may not have been referred to because the key person at each participating site was the TYA lead nurse or clinician. They may have been reflecting on the wider service rather than the role they played within it.

At the time of data collection in the current study, the service specification was a draft. While the clinical community could speculate what was going to be commissioned, the resource for this was not available. The launch of the specification in May 2023 established the Operational Delivery Networks (ODN) [35]. This built on the existing model of care with a centralised specialist TYA unit, linked to the DH, now with the aim of developing joint care models. The DHs were required to be an “active member of the TYA network” to ensure young people were discussed in the TYA-specific multidisciplinary team meeting [34]. The leadership to support this model of care included an ODN manager and lead nurse and physician in each region, and nationally, through the Children and Young People’s Clinical Reference Group as part of the NHS England’s Cancer National Program of Care.

We identified one further barrier to establishing coordinated care: electronic health record systems (EHRS). NHS England’s mandate was for 90% of NHS organisations to have EHRS by 2023, but there was no requirement for this to be standardised across organisations. Warren et al. [51]. examined the impact of multiple EHRS in the UK reporting that “clinicians need the right information about the right patient in the right place at the right time. However, contemporaneous accurate patient information is often not available when it is required” […] “Fragmentation of patient records between multiple health record ‘silos’ has implications for the provision of high quality, cost effective and safe care” [51]. This was reflected in the experiences of healthcare professionals in the current study. While we did not explore this in more depth, it warrants further study to identify mechanisms healthcare professionals implement (officially and unofficially), to ensure there is timely sharing of accurate information across organisations.

D’Amour et al. noted that no dimension took precedence over another; they all needed to be in play, preferably simultaneously for care to be coordinated [17]. D’Amour et al.’s framework helped identify areas of opportunity where the organisation required further strengthening and dimensions where organisational collaboration is optimised. Our findings indicated that the implementation of joint care for TYA cancer care would require leadership. Since the launch of the Service Specification in May 2023, the leadership model is in place, with central coordination through NHS England/Department of Health and Social Care, working alongside leaders locally within each ODN.

Our study has a number of limitations. First, we only obtained healthcare professionals’ perspectives, which are subjective and dependent upon their personal reflections on what worked and did not in their service. If we had been able to observe care as we had originally planned, we would have been able to provide an analysis beyond perceptions and document practices. There were still multiple restrictions in place as a result of the COVID-19 pandemic, so this was not possible. However, healthcare professionals from both PTC and DH were candid about their services so we think we captured unbiased reports of how care was delivered. Second, by only including healthcare professionals, we saw the coordination of care from a single perspective; gaining the young person’s opinion on coordinated care may have illuminated additional issues healthcare professionals were not aware of. Thirdly, three PTCs were not able to participate, and we did not have the perspective of one of the devolved nations. While there were no regional differences in the coordination of care identified in the centres who participated, there is the potential that differences might have existed in non-participating sites, or there were issues unique to these regions that were not captured. Finally, we aimed to recruit ten professionals in each network, but this was not achieved in any region. We encountered difficulties recruiting NHS Trusts as research sites. We had underrepresentation of professionals in DHs and those in the professions outside of medicine and nursing. These populations may have different experiences of communication and coordination to those who participated.

Despite these limitations, our study contributes to the evolving landscape of care network theory by addressing a critical gap between organisational network theory and the practical realities of managing care networks on a daily basis. While traditional organisational network theory offers valuable insights into governance structures, inter-organisational relationships, and network performance, it often overlooks the operational complexity and adaptive behaviours required to coordinate care across diverse settings. By foregrounding the healthcare professionals’ experiences of working in these networks, particularly in navigating fragmented systems, informal routines, and relational work, our findings illuminate the micro-level dynamics that underpin effective care management. This empirical grounding complements and extends existing frameworks (e.g., Braithwaite et al. [52]; van der Weert et al. [53]), suggesting the need for a more integrated theory that bridges strategic design with everyday practice. In doing so, the study advances theoretical understanding of how care networks function in context and offers a foundation for future research into the mechanisms that support sustainable, collaborative care delivery.

## 5. Conclusions

If services are going to be commissioned that require coordinated care, then sufficient resources need to be allocated to be able to deliver these safely. Previously, we showed that establishing a culture of TYA cancer care had a positive impact on patient-reported experience [14], which we suggest has now been established across the UK. D’Amour et al. noted that no dimension took precedence over another; they all needed to be in play, preferably simultaneously for care to be coordinated [17], and our findings indicated that the implementation of joint care for TYA cancer care would require leadership, which was not evident at this time. Since the launch of the Service Specification in May 2023, the leadership model is in place, with central coordination through NHS England. However, effective coordination requires the presence of an outreach team, and these are not in place in all regions across the country. The gold standard would be funding for specialist TYA nurses who have expertise in communicating with young people and experience in cancer to be able to liaise between adult cancer services across their network and the TYA MDT. Pannier et al. recommended the patient navigator (outreach service) as a starting point for TYA services if there was a limited budget because it was a low-cost intervention [52], which could be funded as a cheaper alternative. The implementation of government policy therefore requires government investment to develop this workforce to ensure coordinated care works in practice and operate in an equitable way. Finally, the barrier to coordinated care through EHRS, will hopefully be addressed in the new 10-year Health Plan for England [37], which states a core change in adopting digital technologies to improve efficiency and access. Streamlining communication processes between organisations and between primary and secondary care will facilitate more effective collaboration.

## Figures and Tables

**Table 1:** Dimensions and indicators of collaboration (adapted from D’Amour [17]).

Dimension	Indicator	Description
Shared goals and visions	Goals	Identifying and sharing common goals, particularly the pursuit of patient-centred care, is essential for collaboration, though achieving this goal requires a transformative shift in values and practices.
	Patient-centred orientation	Collaboration often involves diverse and asymmetrical interests, requiring negotiation to align goals; without such negotiation, private interests may dominate, leading to opportunistic behaviour and a loss of focus on patient-centred collaboration.
Internalisation	Mutual acquaintanceship	Developing a sense of belonging and establishing common objectives requires professionals to build personal and professional familiarity through social interaction, which fosters mutual understanding of values, competencies, and approaches to care.
	Trust	Collaboration requires trust in each other’s competencies and responsibilities, as trust reduces uncertainty, but without it, professionals may avoid collaboration, relying instead on outcomes to evaluate and build trust over time.
Formalisation	Formalisation tools	Formalisation clarifies roles and responsibilities through tools like agreements and protocols, but successful collaboration depends more on achieving consensus around these mechanisms than on the level of formalisation itself.
	Information exchange	Effective information exchange through robust systems reduces uncertainty, facilitates patient follow-up, and enables professionals to evaluate partners, playing a key role in building trust.
Governance	Centrality	Centrality involves clear direction from central authorities, with senior managers playing a strategic role in fostering collaboration by formalising processes and agreements across organisations.
	Leadership	Local leadership, whether positional or emergent, is essential for collaboration, requiring shared decision-making and balanced power to ensure all partners contribute and have their voices heard.
	Support for innovation	Collaboration drives innovation by reshaping clinical practices and responsibilities, requiring a complementary learning process supported by internal or external expertise for successful implementation.
	Connectivity	Connectivity ensures individuals and organisations are interconnected, facilitating coordination, continuous adjustments, and problem-solving through systems like information exchanges and committees.

**Table 2:** Summary of inter-organisational collaboration indicators in TYA cancer care in England.

Dimension	Indicator	Evidence of Degree of Collaboration
Shared goals and visions	Goals	• PTCs and DHs wanted to identify a patient quickly and begin the pathway that the specific cancer required. • Both PTCs and DHs wanted to give young people a choice about where to be cared for and preferably use the ‘joint care model’ to accommodate their needs. • There were factors that limited this freedom of choice and hindered early care.
	Patient-centred orientation	• Both PTCs and DHs had a clear common goal of providing patient-centred care. • PTCs and DHs practices and priorities varied, indicating that while there was an intent to be patient-centred, professional and organisational interests still drove decisions of where TYA cancer care was based.
Internalisation	Mutual acquaintanceship	• The involvement in joint clinical activities by PTC and DH was limited. • There were attempts to share information and coordinate care through the TYA outreach roles, but these activities were not consistent or standardised. • There were few structured activities, such as meetings twice a year, that helped professionals get to know each other both personally and professionally. • The need for social interaction and relationship building was described to be important. • Virtual communication (as a result of COVID-19 restrictions) reduced the face-to-face interactions that had previously enhanced relationship building.
	Trust	• Communication between the PTC and DH was not always successful. • Professionals from the PTC held on to the responsibility for their patients as long as possible to avoid “lower quality scans or lab results”. • The frequency of interaction played a role in developing relationships and trust.
Formalisation	Formalisation tools	• TYA cancer care relied on informal processes, experiences, and incomplete information exchange between the PTC and DH, which did not meet young people’s needs or was used inappropriately. • There were formal processes in place to refer patients into a diagnostic pathway and into a PTC, but not for identifying a patient in a DH, transferring them back to the DH, or maintaining communication with the DH during joint care. • Outreach roles and information systems existed, but there was evidence that formalisation mechanisms were still in the process of being negotiated or constructed (i.e., honorary contracts or IT access). • Some professionals were still unsure about who did what.
	Information exchange	• There were efforts to enhance information exchange through MDTs, shared IT systems and communication channels. • IT was not standardised across organisations nor always accessible. • Issues with accessing patient notes on electronic health record systems and lack of integration between children and adult data systems, highlighted gaps in the information exchange infrastructure. • Professionals faced challenges in information exchange, and difficulties in getting timely updates and feedback contributed to ongoing issues with collaboration and trust-building. • The infrastructure for information exchange did not fully meet the needs of all professionals involved in the care continuum in the NHS and excluded those working in some of the charities.
Governance	Centrality	• No explicit mention of senior roles or central authorities. • No evidence of a political or strategic role that would support and drive collaborative processes. • Absence of central leadership resulted in a lack of clear, coordinated direction for collaboration
	Leadership	• There was no strong indication of a unified leadership structure that effectively drove collaboration between PTC and DH. • Leadership was dispersed, with various stakeholders potentially exercising leadership either at PTC or at DH level, but not throughout the care network. • Fragmentation of leadership led to inefficiencies and a lack of clear direction.
	Support for innovation	• Both PTC and DH engaged in innovative practices and adjustments, such as increasing mental health support, setting up shared IT systems, creating new outreach roles or transition roles, and planning for more consistent communication (e.g., weekly virtual meetings). • These efforts indicated recognition of the need for innovation to improve care coordination and patient outcomes. • Gaps in current expertise and resources were highlighted, including the need for more specialised roles, such as TYA physiotherapists and inpatient nurses. • There was a desire for increased funding for peer support and overall NHS capacity.
	Connectivity	• There was space for discussion and connectivity, such as MDT meetings and occasional feedback meetings, particularly through outreach roles. • The venues for discussion (virtual or physical) and feedback were not consistently accessible either due to individual or clinical commitments. • Virtual communication amid COVID-19 pandemic restrictions decreased face-to-face interactions between health professionals, negatively impacting the quality and frequency of their encounters. • There were other barriers for patient discussion, e.g., clashes with local meetings, non-representation of DH staff in the TYA MDT, lack of a named consultant, or lack of an outreach nurse. • There were ad hoc opportunities for connectivity in some instances, but it was not systematic and widespread across regions

DH: designated hospital; IT: information technology; MDT: multidisciplinary team; NHS: National Health Service; PTC: principal treatment centre; TYA: teenage and young adult.

## Data Availability

All relevant data for this study are included in the manuscript.

## Supplementary Materials

The following supporting information can be downloaded at: https://www.mdpi.com/article/10.3390/cancers17233874/s1, Interview topic guide.
