# Supplementary material for: Exploring the Coordination of Cancer Care for Teenagers and Young Adults in England and Wales: BRIGHTLIGHT_2021 Rapid Qualitative Study
Source: Cancers (Basel). 2025 Dec 3;17(23):3874. doi: 10.3390/cancers17233874 (PMC12691236; doi:10.3390/cancers17233874)
Supplement: Supplementary file 1 [file cancers-17-03874-s001.zip › cancers-3871809-supplementary.pdf]

Supplemental file: Interview topic guide

1. Can you tell me about your current role?
2. How long have you been in this role?
3. How long have you been delivering care to children and young people with cancer?
4. Can you tell me how care delivery is organised at this hospital? Can you guide me through the steps/stages in the pathway?
    a. Are there any deviations from this pathway? If so, why do these occur?
    b. Has the pathway changed?
    c. Are there any planned changes in the near future? If so, why are you planning to make these changes?
5. Does care delivery for young patients span this hospital? This Trust? Can you tell me about the different places where children might receive care along the pathway we discussed? How do patients navigate through these settings? Are there any problems you have identified?
6. Who are the members of the clinical team that the children might come into contact with? (this hospital, the Trust, other settings)
7. Do you interact with staff members from these other settings? If so, how do you share information?
8. Is there any joint decision-making with staff from other sites? If so, how is this facilitated? Are there any barriers to joint decision-making?
9. Do you think the hospital offers a ‘joint care’ to patients?
   If so,
    a. How would you define joint care?
    b. How is this joint care carried out in practice?
    c. What are the factors acting as barriers in the delivery of joint care?
    d. What are the factors acting as enablers in the delivery of joint care?
    e. Are there any plans to make changes in joint care models?
10. If not,
    a. How would you define joint care?
    b. Why do you think this site does not offer ‘joint care’?
    c. Have there been previous attempts to deliver joint care?
    d. Are there any factors that have acted as barriers in these attempts to deliver joint care?
    e. Are there any plans to deliver joint care in the future?
11. What are the most important aspects of the care this site delivers to young people?
12. What do you do well?
13. What do you think needs to be improved?
14. Is there anything else that you think we should know that I have not asked you?
